# The Actions of the Heart

**Published:** 1865-04

**Authors:** 


### THE ACTIONS OF THE HEART.

Will venous blood stimulate the actions of the left auricle and ventricle of the heart? I believe it will not, or at least very imperfectly. In every circuit which the blood makes, when it reaches the pulmonary arteries it is loaded with the recrement, or the products of the decay of the body. When in the pulmonary arteries, this decay, partly by a chemical and partly by a mechanical operation, is converted into gas, and expired out of the system in the shape of impure air. When in the pulmonary veins, the blood is further revivified by the inspiration of atmospherical air, and when here, again, partly by a chemical and partly by a mechanical combination, heat is evolved. In this state the blood enters the left portals of the heart, and thus purified is well qualified to carry on the circulation, and at the same time to maintain the heat of the body. It may be asked, how is the venous blood circulated through the right auricle of the heart? I maintain that this is done by suction; the blood is thus drawn from the cavas to the right portals of the heart, and thence to the pulmonary arteries.

I am, Sir, yours, etc. PHYSIOLOGIST.

*Liverpool*, 1865. [*Lancet.*

